# Cultured fish epithelial cells are a source of alarm substance

**DOI:** 10.1016/j.mex.2017.11.003

**Published:** 2017-11-11

**Authors:** Heather A. Hintz, Courtney Weihing, Rachel Bayer, David Lonzarich, Winnifred Bryant

**Affiliations:** University of Wisconsin Eau Claire, United States

**Keywords:** Non-invasive harvest of alarm substance in Ostariophysi, Alarm substance, Primary cell culture, Predator avoidance, Darting, Creek chub

## Abstract

In various species of fishes, the importance of visual cues in the determination of environmental threat and subsequent predator avoidance is clear. Chemical cues also play an essential role facilitating predator avoidance. Among fish in the superorder Ostariophysi, club cells in the epidermis produce an alarm substance. Damage to the skin during a predation event releases an alarm substance (AS), which diffuses through the water column and binds to olfactory receptors of conspecifics. Fish then engage in a number of anti-predator behaviors that may include darting, schooling, or hiding.

Behavioral responses to AS and physiological mechanisms that underlie those responses is an active area of study. However, because the precise chemical composition of the alarm substance is unknown, AS is not commercially available. Thus, when fish are challenged alarm substance in various experiments and assays it is obtained from skin extracts or via perfusion of shallow cuts in the epidermis. Both procedures are effective but require the animal to be sacrificed.

In this manuscript, we report:

• A non-invasive primary cell culture protocol to obtain alarm substance and does not require the model organism to be killed.
• The demonstration of anti-predatory behaviors in fish exposed to alarm substance collected by this method.

## Graphical abstract

**Figure:**
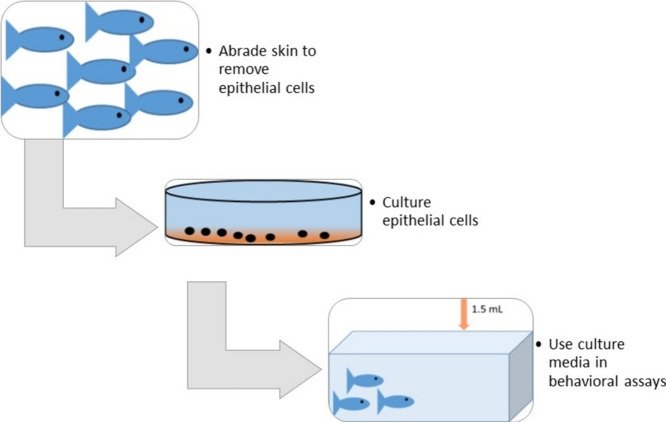

## Method details

### Animals and housing

Sexually immature (40–70 mm in length) male and female creek chub (*Semotilus atromaculatus*) were used for these studies as they have been observed to express more club cells than adult. Given their abundance and accessibility, the animals were wild caught. Fish were housed in glass aquaria at 20C and maintained on a 12:12 light cycle. Food pellets were provided daily. Filters and aeration maintained water quality and fish were visually inspected daily for normal behavior and obvious signs of infection.

### Cell collection

All reagents should be used at room temperature. Remove fish from the home tank and place in 1.5 L of water containing 400 μL of an oil of cloves solution (1:10 clove oil in 95% ethanol (should yield a concentration of 100 mg/L) (store in a dark bottle as solution is light sensitive). The solution should induce a light plane of anesthesia in fish in approximately 5 min. When the fish is unable to right itself in the water and/or does not try to avoid handling, collection can begin. Place the fish on its ventral side on a wet paper towel and gently abrade the epidermis with a sterile scalpel blade. Focus cell collection on the anterior end of the animal and avoid removal of scales. Place epidermal cells in a 15 mL polypropylene conical vial containing 10 mL room temperature phosphate buffered saline (PBS) (no calcium or magnesium) (Life Sciences) supplemented with 1.5% penicillin/streptavidin solution (10,000 IU/mL, GIbco) and 1.5% Fungizone ^®^ (antimycotic, Gibco), 1.5% kanamycin (Gibco), and 1.5% tetracycline (Gibco) (this solution will be referred to as supplemented PBS). Sample collection should take less than 60 s. Immediately afterwards, place the fish back into its home tank and observe for normal behaviors. Recovery from anesthesia should occur within minutes. Pool cells from six fish.

### Primary cell culture

**Fig. 1:**
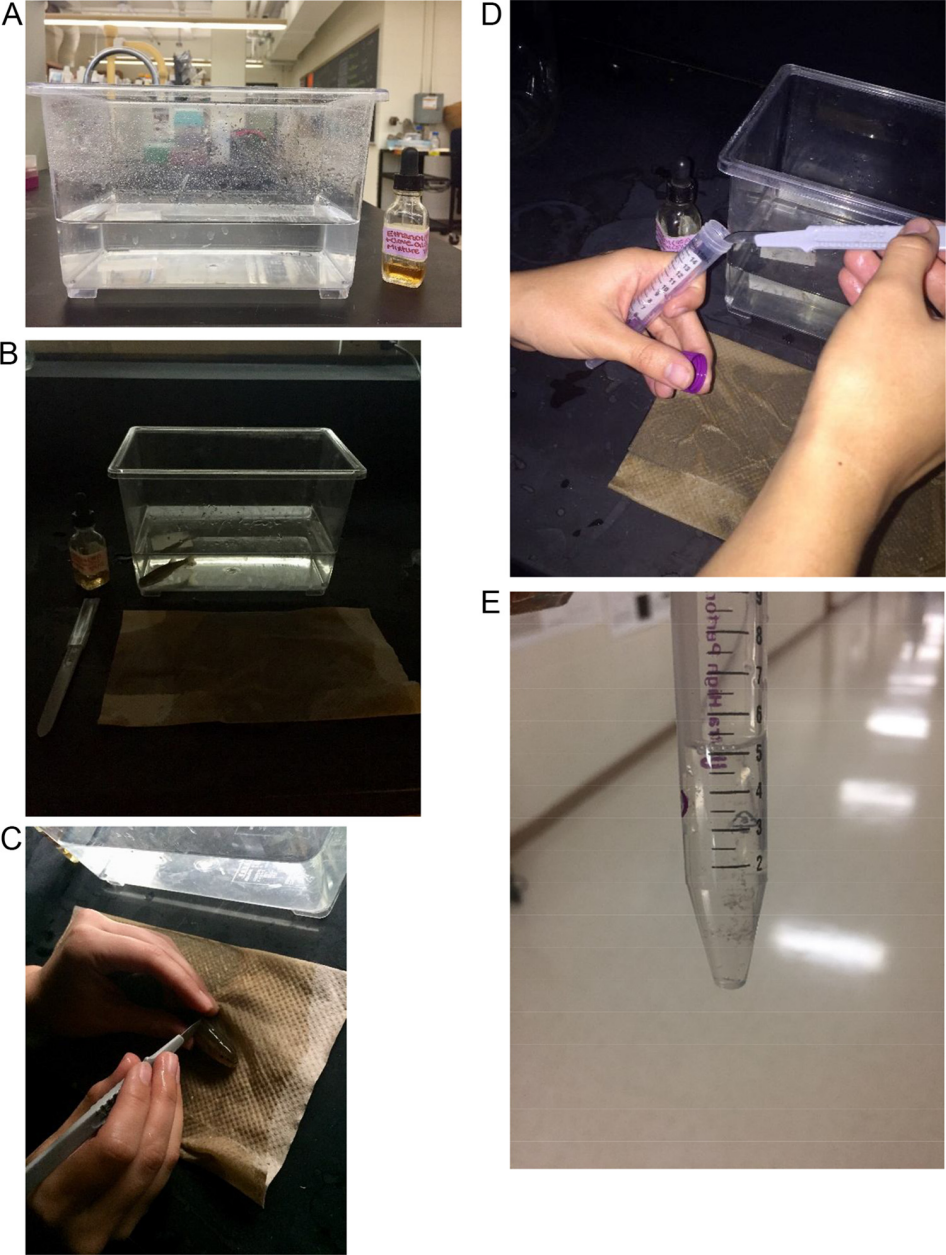
Collection of epithelial cells for primary culture. Fish were removed from home tanks and place in ∼1500 mL of water containing 400 uL of oil of cloves solution (Fig. 1A, B). After a light plane of anethesia was induced, epithelial cells were removed from the fish with a clean scalpel (Fig. 1C) and placed into a 15 mL polypropelene vial containing supplemented PBS (Fig. 1D, E).

All reagents should be used at room temperature (Fig. 1). Vortex the conical vial containing cells for approximately 15 s at the highest speed (speed 10 using a VWR Mini Vortexer VM3000) to assist is disrupting the mucus that was collected with the cells. Centrifuge the cells 5000 rpm for 5 min. Remove the supernatant and discard. Add 10.0 mL fresh supplemented PBS. Repeat the vortex/wash 2X. After the final wash, remove as much PBS as possible without disrupting the pellet of cells in the bottom of the tube.

Resuspend the final pellet in 6.0 mL Leibovitz's L-15 culture medium supplemented with 20% fetal bovine serum, 1.5% penicillin/streptavidin and 1.% Fungizone, 1.5% kanamycin and 1.5% tetracycline (this solution will be referred to as supplemented Leibovitz's). Leibovitz's medium was selected for cell culture because it is CO_2_ independent; thus, cells require no O_2_ or CO_2_ supplementation. This media also maintains physiological pH through salts, high concentration of basic amino acids and galactose. and vortex. If scales are evident in the tube, allow the tube to stand vertically for 15 s—the scales will settle to the bottom. Avoiding the scales if present, remove the media/cell solution from the conical vial with a sterile Pasteur pipet and transfer to a sterile T25 flask with a vented cap (Corning).

Culture T25 flasks at room temperature on a clean lab bench. In 24 h, pellet the cell suspensions at 5000 rpm for 5 min. The supernatant (containing culture media and cellular products) should immediately be stored at −20C in a 15.0 mL polypropylene conical vial for use in behavioral assays.

Add 10.0 mL of supplemented PBS to the pellet and vortex for 10 s at max speed to resuspend. Centrifuge the cells 5000 rpm for 5 min. Remove the supernatant and discard. Add 10.0 mL fresh supplemented PBS. Repeat the vortex/wash 2X. After the final wash, remove as much PBS as possible without disrupting the pellet of cells in the bottom of the tube. After the third wash, resuspend cells in 6.0 mL supplemented Leibovitz's.

Maintain primary cell culture and harvest media every 24 h for a maximum of 5 days.

For use in behavioral assays, thaw frozen media in conical vials on ice. Warm the vial to room temperature with hands just prior to use.

### Behavioral assay

**Fig. 2:**
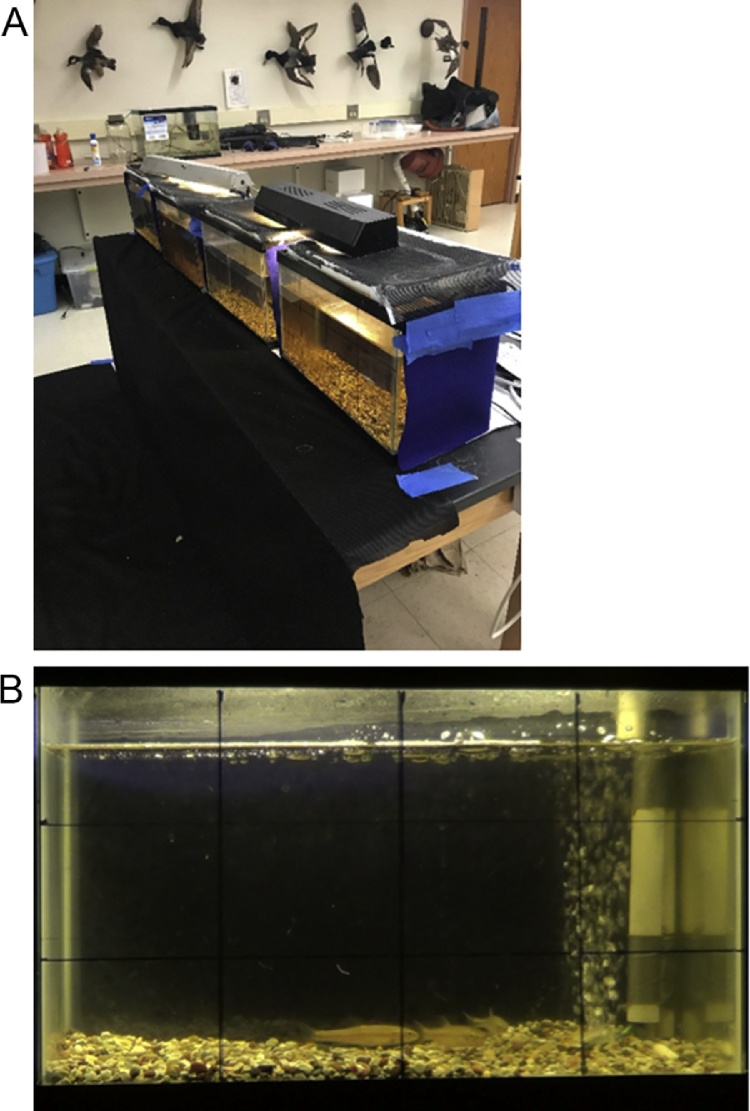
Experimental set-up and behavioral assays. Behavioral trials took place under low light conditions—dark cloths were used to minimize the transmission and reflection of light (2A). Behavioral experiments took place in aerated tanks; 3 fish were tested per trial (2B).

For behavioral assays, sexually immature creek chub were transferred to a room with low levels of light—windows and white floors were covered with black cloth to minimize transmission and reflection of light, respectively. Fish were matched for age by size and 3 fish were placed in an aerated glass aquarium. Their activity was digitally recorded with a Sony HDR-CX210 camcorder (Fig. 2).

Fish were recorded in the untreated environment for 3 min, in the presence of a control solution (distilled water) for 3 min, and finally, in the presence of 1.5 mL media, which was taken from epithelial cells after 24 h of culture (Fig. 2). Seconds of darting behavior by each fish under each condition was calculated from the digital record. In our model, darting was the most robust antipredatory behavior observed. Different species may exhibit other behaviors more prominently. Therefore, quantify the most consistently expressed antipredatory behavior. Darting behavior for each fish was measured; nine trials were completed. Data were analyzed using a *t*-test in GraphPad Prism. p values of ≤0.05 were considered statistically significant.

**Fig. 3:**
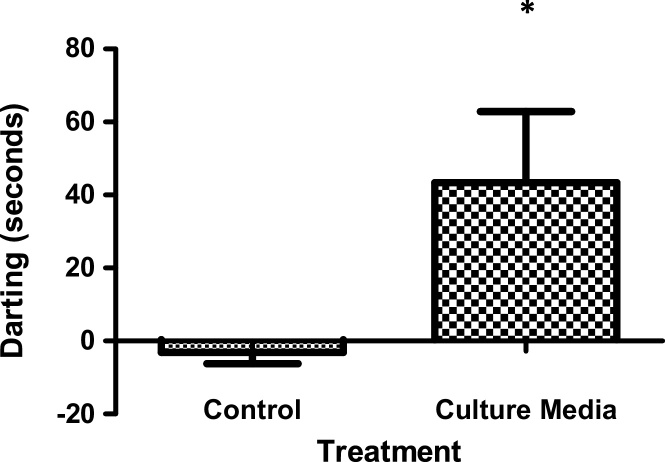
Primary cell culture media induces darting behavior in Creek Chub. Juvenile Creek Chub were observed in the absence of treatment, in the presence of a control solution (distilled water), and in the presence of 1.5 mL media from cultured epithelial cells (after 24 h). Observation time was 180 s. Seconds of darting behavior was averaged for 9 trials; means were compared using a *t*-test. *, significantly different from control, p < 0.05.

Darting behavior in fish was significantly increased following addition of cell culture media as compared to darting behavior after addition of distilled water (Fig. 3). Responses were acute, beginning immediately after addition of the media to the tank and lasting approximately 40 s.

## Additional information

Vision is a key strategy by which individual aquatic species avoid predators. However, in low-light or turbid conditions the injury-induced release of chemical messengers play a key in allowing an individual to communicate threat to conspecifics (reviewed by [1]). Among fish in the superorder Ostariophysi, anatomically distinct club cells in the epidermis produce an alarm substance, which is released if those cells are damaged (reviewed by [2], [3]). The alarm substance then binds to olfactory receptors of conspecifics, resulting in a range of anti-predatory behaviors (including freezing, darting, schooling, feeding, and hiding) which may persist for up to 4 h after withdrawal of the alarm substance [4]. The precise composition of the alarm substance is unknown and thus, a purified preparation of alarm substance is not commercially available. However, determining the precise composition of alarm substance is an active area of study [5], [6]. Most recently, chondroitin and hypoxanthine 3-*N*-oxide have been shown to induce anti-predatory behaviors in fish [7], [8]. This suggests that alarm substance contains these compounds as active ingredients or other chemicals that are similar in structure. In bioassays, fish epithelium is usually used as a “natural” source of alarm substance. A prescribed section of skin is removed and homogenized in water; fish exposed to the solution exhibit anti-predator behavior [7], [10]. Alternately, shallow incisions are made in the epidermis and flushed with water; this solution induces anti-predator behavior as well [9]. The aforementioned procedures are effective at extracting alarm substance but the fish from which they are obtained must be killed.

*Understanding behavioral responses to alarm substance in fishes is significant because they represent a first step in understanding the neural and physiological mechanisms that mediate fear and anxiety in humans*. The method we present in this manuscript allows for the development of a sustainable source of alarm substance for use in chemical characterization and bioassays without killing the research model. Our method for collecting epithelial cells is inexpensive, non-invasive, and allows for fast recovery of the experimental model. Additionally, cells can be collected from the same fish multiple times. We also demonstrate that our source of alarm substance significantly increases darting behavior in wild caught Creek Chub minnows (*Semotilus atromaculatus)*.
